# Routine performance and errors of 454 HLA exon sequencing in diagnostics

**DOI:** 10.1186/1471-2105-14-176

**Published:** 2013-06-03

**Authors:** Norbert Niklas, Johannes Pröll, Martin Danzer, Stephanie Stabentheiner, Katja Hofer, Christian Gabriel

**Affiliations:** 1 Red Cross Transfusion Service for Upper Austria, Krankenhausstraße 7, 4017 Linz, Austria

**Keywords:** Next-generation sequencing, Human leukocyte antigen typing, Error characteristics, Quality control

## Abstract

**Background:**

Next-generation sequencing (NGS) has changed genomics significantly. More and more applications strive for sequencing with different platforms. Now, in 2012, after a decade of development and evolution, NGS has been accepted for a variety of research fields. Determination of sequencing errors is essential in order to follow next-generation sequencing beyond research use only. This study describes the overall 454 system performance of using multiple GS Junior runs with an in-house established and validated diagnostic assay for human leukocyte antigen (HLA) exon sequencing. Based on this data, we extracted, evaluated and characterized errors and variants of 60 HLA loci per run with respect to their adjacencies.

**Results:**

We determined an overall error rate of 0.18% in a total of 118,484,408 bases. 31.3% of all reads analyzed (n=349,503) contain one or more errors. The largest group are deletions that account for 50% of the errors. Incorrect bases are not distributed equally along sequences and tend to be more frequent at sequence ends. Certain sequence positions in the middle or at the beginning of the read accumulate errors. Typically, the corresponding quality score at the actual error position is lower than the adjacent scores.

**Conclusions:**

Here we present the first error assessment in a human next-generation sequencing diagnostics assay in an amplicon sequencing approach. Improvements of sequence quality and error rate that have been made over the years are evident and it is shown that both have now reached a level where diagnostic applications become feasible. Our presented data are better than previously published error rates and we can confirm and quantify the often described relation of homopolymers and errors. Nevertheless, a certain depth of coverage is needed, in particular with challenging areas of the sequencing target. Furthermore, the usage of error correcting tools is not essential but might contribute towards the capacity and efficiency of a sequencing run.

## Background

Next-generation sequencing systems have boosted genetics in the last few years. The reduction of costs, wet-lab workflow complexity and the gain of read length has led to an enormous increase in sequencing projects and sequencing data [1]. Roche/454 Life Sciences is one of the major players in the NGS field as their technology of pyrosequencing allows for the longest possible reads of all 2^nd^ generation sequencing techniques with further technological improvements proposed, moreover, two different sized platforms allow for scalability [2]. This technology is based on DNA templates immobilized on beads which are loaded onto a PicoTiterPlate (PTP). Subsequently, nucleotides flow over this plate in periodic cycles and get incorporated if complementary to the template strand. An enzyme cascade is activated, leading to the release of photons. These photons are detected by an ultra-sensitive CCD camera. Lengths of homopolymers (stretch of the same nucleotides) are determined by the amount of emitted light [3], especially long homopolymers are a huge challenge of the 454 technology itself, bioinformatics and analysis respectively interpretation [4,5].

It is a logical consequence to follow NGS from the basic research applications to routine diagnostic assays [6-8]. Using NGS for human leukocyte antigen (HLA) typing is one of the most evolving fields of application and pushing forward for routine diagnostics [9-13]. Our lab is certified by the European Federation for Immunogenetics for HLA typing and has years of experience in HLA typing and next-generation sequencing [14,15]. For transplantation of haematopoietic stem cells DNA based, high-resolution typing of HLA is an absolute necessity in order to gain a best possible histocompatibility to reduce the risk of a severe graft-versus-host-disease [16]. Most recently, we have demonstrated NGS HLA typing as feasible for routine diagnostics [17].

For diagnostic applications it is essential to know possible errors in workflow and data analysis. There are already implemented mechanisms controlling and dealing with errors in a quality management controlled laboratory. Every next-generation sequencing platform and technique has its own application dependent error profile. Several groups have estimated errors for special fields of genomics, including bacterial, viral and antibody sequencing [4,18,19].

Here we present a detailed error assessment for sequences of NGS HLA typing on a 454 platform. We analyzed multiple runs and point out the level of safety for diagnostics NGS applications on the basis of error occurrences and if any of them are recurring and linked to sequence motifs.

## Results

### Performance, accuracy and errors

Taking all six runs together, 373,792 reads passed built in quality filtering [20], with a total of 146,860,970 bases sequenced and average read length of 393 base pairs.

Raw run performances (before trimming and further analysis) of the six runs are shown in Table 1, together with filter metrics and read statistics.

**Table 1:** Overall run performances

**Run**		**1**	**2**	**3**	**4**	**5**	**6**
Passed Filter	[reads]	58,303	58,230	59,991	70,988	70,477	55,803
Short	[%]	48.97%	51.79%	45.68%	44.29%	42.92%	54.74%
Qual 98% 400 bp	[%]	82.68	80.61	85.18	76.49	82.37	75.84
Control	[wells]	5,688	4,626	5,701	5,117	7,471	6,084
Raw	[wells]	169,261	187,287	163,378	167,569	180,379	187,583
Key pass	[wells]	161,395	180,717	155,583	160,344	170,507	178,334
Median Read length	[bp]	417	418	424	414	316	406
Avg Read length	[bp]	408.94	410.24	411.66	402.70	349.29	379.03
mixed	[%]	10.89%	11.36%	10.49%	7.73%	10.85%	10.80%
dot	[%]	3.82%	4.43%	5.04%	3.53%	4.69%	2.87%
Bases	[Mb]	24.31	24.34	25.44	29.39	22.27	22.66

Raw run performance of six runs is shown.

93.5% of the generated raw reads could be aligned to HLA reference sequences and were used for further analysis. After trimming primers and reducing reads to exon information, 118,484,408 bases (81% of the original output) were taken into account when calling variants and determining errors. 563 variants in the exon regions were defined as true variants, known by Sanger sequence based typing (SBT) and additional pseudogen analysis. Besides, 13,505 variants were detected and categorized as errors.

109,473 reads had at least one error, therefore 31.3% of all reads contain errors in their coding region and on average one read had 2.08 errors. After applying the error correction tool Acacia, errors still remained in 25.1% of all reads [21].

The number of reads containing one error was multiplied with the corresponding length of the error resulting in 212,415 bases being erroneous. The total error rate of 0.18% was defined by the percentage of wrong bases in the number of total exon bases, where insertions account for 0.09%, deletions for 0.04% and substitutions for 0.05%. Insertions had an average length of 1.12 bases, deletions 1.07 bases and substitutions one base; summarized, errors had a length of 1.06 bases.

38.15% of these errors were detected in all six runs, meaning 0.07% reproducible errors (0.03% insertions, 0.03% deletions and 0.008% substitutions) associated with 81,026 bases.

### Quality scores

**Figure 1:**
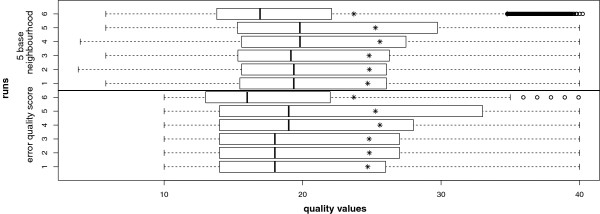
**Base quality scores at error positions.** Quality scores of bases were collected at error positions and plotted in boxplot format (quartiles) per run, compared with the quality scores of a five base neighborhood surrounding the error position. Base quality scores of the neighborhood show a tendency of higher values than the actual error position. Runs 1–3 were performed with the same bead pool, the quality scores of these three runs show no significant differences, runs 5 and 6 were enriched manually and differ significantly. The asterisks mark the overall quality of the particular sequencing run.

Average quality score (phred equivalent quality scores, Q = -10 * log_10_(error rate), estimated by the GS Junior software [20,22]) of all sequenced bases (taken the six runs together) is 35.39; counting only bases used for HLA typing average quality score is 35.46. Average quality score of error positions was 16.08, meaning an accuracy (calculated from GS quality values) of 97.53%, quality score is less than or equal to 25 at 73.1% of incorrect bases. The adjacent base quality scores of a neighborhood of 5 bases averaged was precisely higher at 17.00 (98.00% as base accuracy, calculated from GS quality values). Boxplot of quality scores in Figure 1 compares the six runs on error position and five base average, quality scores of runs 4 and 5 have slightly better quality scores on both parts.

### Homopolymers

**Figure 2:**
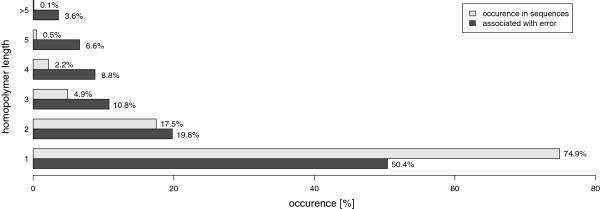
**Homopolymer associated errors.** Error positions were tested as to whether or not they are located at a homopolymer position (no homopolymer: length = 1) and to which percent different lengths are affected (dark gray). Reference sequences were scanned for homopolymer occurrences and percentages of hompolymer lengths were plotted next to error association (light gray). Homopolymers with lengths greater than five were combined. Percent values are rounded.

**Figure 3:**
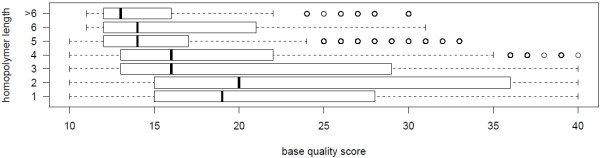
**Base quality scores of erroneous homopolymers.** Base quality scores of homopolymers showing erroneous bases as boxplots (quartiles) allowing the comparison of accuracy of homopolymers with different lengths. Homopolymers with lengths greater than six were summarized.

50.4% of errors were outside of a homopolymer region, 29.8% were adjacent to a n-mer of length 3 or longer. Figure 2 displays portion of homopolymer lengths associated with errors compared to the percentages of homopolymers in analyzed sequences with given length. Correlation of homopolymer’s length and quality values is -0.195 which is highly significant (p<0.001), Figure 3 illustrates correlation with boxplots where base quality scores decrease with the length of homopolymers.

### Read position

**Figure 4:**
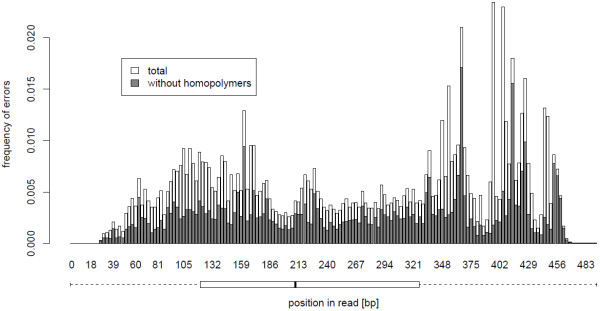
**Number of errors at read position.** Frequency of errors occurring at a specified read position (in relation to the coverage) in total (white) and without homopolymer association (gray). Bars are unstacked, homopolymer proportions plotted to the front. The boxplot below displays the quartiles of errors across read positions (total). Errors at the first positions are very infrequent, furthermore, this section of sequence is located outside the analyzed exon.

**Figure 5:**
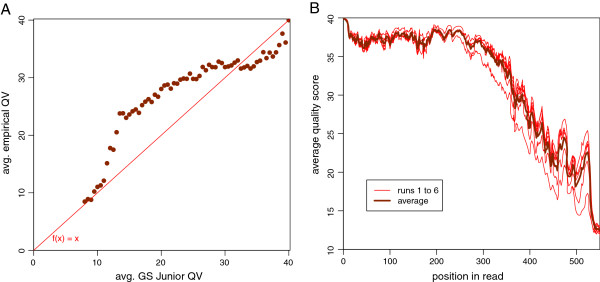
**Average base quality scores. A**. The estimated quality values produced by the GS Junior were compared to the empirical quality values calculated by error frequencies. The thin red line marks the ideal line if the system would estimate all values correctly. **B**. Development of base quality scores (averaged per position) over the reads’ lengths (and run progress). Comparison of six runs (thin red lines) and their average (bold dark red line). The quality drop at the first 20 bases is no application specific phenomenon but explained by the mathematical calculation of the quality scores and their dependence on previous flows and bases.

Taking into account errors adjacent to homopolymeric regions, there is no significant peak in the distribution along the read distance. The distributions along the read positions regarding specific amplicons corresponding to HLA exons are given in Additional file 1. Homopolymeric associated errors have no significant effect on these positions. Figure 4 shows the distribution of errors over the read length. Base quality over the read lengths respectively the progress of a run is plotted in Figure 5, a characteristic development over sequence length is apparent.

## Discussion

### Run performances

Several publications analyze accuracy and errors in 454 sequencing data. Huse et al. [4] analyzed bacterial 16S rDNA with the older GS20 platform and affirmed their basic foundings for Standard chemistry [23], Prabakaran et al. [19] characterized errors in a small portion of 3,467 antibody sequences and Gilles et al. [24] used control DNA fragments of the 454 workflow for error assessment. As stated previously [24], error characteristics is sequence motive dependant, hence every application needs its own error profile.

Run performance of the GS Junior platform is stated to be approximately 136,760 reads per run for shotgun sequencing [2]. 70,000 reads are expected from amplicon experiments [25], most of our runs in this study do not reach this number of sequences, resulting in average 62,299 reads, however, being sufficient for HLA genotyping of 10 samples (six loci per sample).

### Per base error rates

The used enzyme for amplification has an error rate of 8.3×10^-6^[26]. Accordingly, approximately 25,052 erroneous bases in our experiment are due to PCR artifacts. These bases contribute 11.8% to our total error rate. Our error rate of 0.18% differs significantly from already published error rates: 0.49% Standard chemistry [4], 0.4% and 1.07% for Titanium chemistry [24,27]. The high error rate of 1.07% can be explained through the use of the 454 control fragments for error analysis. Considering (long) homopolymers being the weak point of 454 systems, they are overrepresented in the control fragments in contrast to natural DNA sequences. In Lind et al. an error rate of 1.1% for a shotgun HLA sequencing approach is given, sequenced with Standard chemistry [28]. Since GS20 many improvements in protocol, reagents and software have been made to the 454 technology. Additionally, reads tend to become error prone towards their end [24], the (intron) trimmed analysis furthermore reduces possible errors due to errors being rather located at the reads’ ends. Due to this analysis strategy, 19% of the produced output is not analyzed.

Insertions (50%) are the most frequent errors followed by substitutions (28%) and deletions (22%), the substitution rate is even lower than for Illumina’s MiSeq system stated in Loman et al. [2]. Both publications mention insertions as the most frequent errors. In contrast to previously published error data substitutions account for the second frequent errors, including PCR or application specific errors. Gilles et al. reported a seven times lower substitution rate than deletions originating from the overrepresented homopolymers.

68.7% of all reads were free from errors, consistent with Huse et al. [4]. Hence, without denoising [21] or smoothing [18] a loss of one third of data must be taken into account. With error correction additional 6.2% of reads (of total reads generated) could be recovered, resulting in a quarter of sequences still exhibiting errors. We use a conservative approach without additional modifications of the data to prevent introduction of false positive mutations. The majority of reads containing errors (77.2%) has less than three wrong bases. The reduced error rate in our setting is the reason for the satisfying average error per read rate of 2.08 errors and the average length of 1.06 bases per error.

For 1,743 variants (13%) there was evidence (in at least one of the six runs) supporting the mutation in both sequencing directions, in accordance with Challis et al. [29].

### Read position and motifs

The occurrence of erroneous bases was highly connected to read respectively reference position, 38.15% of them occurred at the same positions when resequencing. There is strong evidence that errors are also highly linked to special sequence positions and DNA patterns. As a result the individual error rates of the six runs only slightly differ from each other respectively the given average values. Vandenbroucke et al. indicated that every amplicon has its own error profile [30].

Based on our examination we can state that more errors are located in the second half of the read than in the other half, indicated by a median error position of 236 with an average read length of 393.

### Quality scores

Quality values calculated from the averaged error rates were compared to the average quality values estimated by the GS Junior at the same positions (Figure 5A). Below values of 30, the empirical rate is higher than the estimated value; above 30 the GS Junior overestimates its own performance (Q30 = accuracy of 99.9%).

The distribution of quality scores along the read distance (Figure 5B) of all runs exhibits a very equal pattern, showing that some regions have valleys (lower quality scores) while others have peaks (high quality scores). The overall pattern with a considerable decrease at around 300 bp is typical for all GS Junior runs; positions and power of peaks are library specific and highly reproducible. The quality scores of surrounding error positions correspond to the overall run performance that was slightly better in run 4 and 5 and below expectations for run 6 due to variations of the complex workflow and chemistry.

Comparing the quality values of the actual error position to their neighborhood (see Figure 1) reveals that the erroneous base is represented by a quality valley. Figure 1 reveals that quality values of areas of errors are below other positions, the actual error position is even lower.

### Homopolymers

Homopolymers form a major challenge in base calling algorithms in the 454 sequencing systems, thus, errors turning up are highly connected to homopolymer regions [4,24]. On a first glance 50.4% of errors outside homopolymeric regions may seem contrary. Considering the distribution of homopolymers with given lengths in the reference sequences for HLA, it is significant (p<0.01) that homopolymers are more attractive to form errors than single bases (proportions are plotted in Figure 2). The length of homopolymers correlates with a decrease of accuracy drops in general, with the exception of 2-mers having the best quality scores at error positions, displayed in Figure 3.

## Conclusions

In this study we present a detailed error characterization of 454 sequencing using data from a diagnostic assay. In our amplicon sequencing approach exactly 0.18% of total bases used for HLA typing are erroneous. This error rate supports and allows the benefit of typing HLA with 454 next generation sequencing. Although amplicon sequencing is considered as more sophisticated than shotgun from a bioinformatics perspective [27], the presented data are even better than previously published shotgun approaches [28].

Several software products are able to correct errors, however most of them are specialized on a specific application and sequence context. Moreover, if error models are already known, many tools are able to simulate sequencing data with a reference sequence but without taking neighboring sequence motifs into account [31-33].

Additionally, knowing error rates allows for the reduction of sequence depth needed for a certain accuracy [34], furthermore allowing diagnostics to be more cost-effective. The given data outperforms previous publications using test fragments, non human samples or outdated software or reagents.

## Methods

### Clinical setting and experimental design

**Figure 6:**
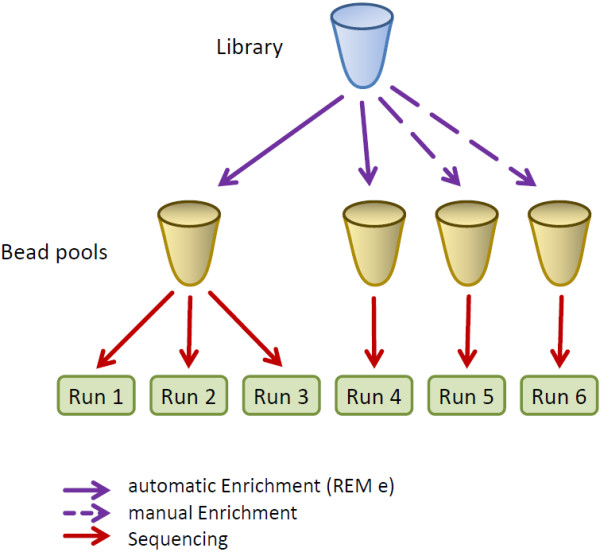
**Experimental setup.** One library was created and used for all six runs, for run 1–3 beads emPCR and enrichment was performed together, hence they use the same bead pool. Beads for run 4 were prepared separately. Runs 1–4 use automatic enrichment on a liquid handling robot together with the REM e. Beads for run 5 and 6 were generated independently and enrichment was performed manually.

Genomic DNA used for GS Junior sequencing originates from routine HLA typing for haematopoietic stem cell transplantations. Ten typical Caucasian samples were randomly selected for a detailed analysis of sequencing performance. Specimens were collected after signing a written consent for sequence based HLA typing. For this particular study an approval by an ethic committee was not required. It was a technical study with no impact on patients or their treatment. Genomic DNA was isolated with an automated DNA isolation system (MagnaPure Compact, Roche Diagnostics, Mannheim, Germany), followed by amplification of 17 amplicons for six loci of HLA typing with Expand High Fidelity PCR System (Roche Diagnostics, Penzberg, Germany) and automated purification and pooling with a Hamilton Microlab STAR (Hamilton Robotics GmbH, Martinsried, Germany). The created pool was independently sequenced six times. Emulsion-PCR and bead recovery were performed according to supplier’s instructions (Roche 454 Life Sciences, Branford, USA). Automated enrichment with REM e (Roche 454 Life Sciences, Branford, USA) was used for enrichment of beads in runs 1 to 4, implemented on a Hamilton Microlab STARlet (Hamilton Robotics GmbH, Martinsried, Germany), magnet time 80 sec, 12 wash steps. Enrichment for runs 5 and 6 was performed manually. Runs 1 to 3 were sequenced with beads from the same bead pool (see Figure 6). Sequencing with GS Junior system, Titanium chemistry (Roche 454 Life Sciences, Branford, USA) was done following the manufacturer’s instructions without modifications. So, we do not compare library preparation but intrinsic 454 sequencing performance irrespective of sample DNA quality, PCR amplification bias and general library preparation issues.

### Data analysis

Data processing was carried out on the GS Junior attendant PC with default settings for Amplicon sequencing without any modifications to processing pipeline or filtering. HLA genotypes are routinely typed with ATF software (Conexio Genomics, Perth, Australia). For assessment of variations and errors the GS Amplicon Variant Analyzer (AVA) (Roche 454 Life Sciences, Branford, USA) was used for alignment and output of sequences.

### Variant and error detection

Genotypes of tested samples were determined beforehand by Sanger SBT. Therefore expected variants could be defined with an allele database (IMGT/HLA 3.7.0 2012–07) [35]. To overcome missing intron information in the allele database only exon sequence was considered. In principle, AVA software does not output all detected variants by default. Therefore variants were generated by a Perl script (Roche 454 Life Sciences, Branford, CT, USA) going through all multiple alignments in AVA and reporting discrepancies from the reference sequences. Sequences A*01:01:01:01, B*07:02:01, C*01:02:01, DQB1*02:01:01, DRB1*01:01:01 and DPB1*01:01:01 were used as references.

Detected variants were compared to known variants. For locus A, exon 2 the pseudogen HLA-Y is amplified by approximately 25%, for locus DRB1 the loci DRB3, DRB4 and DRB5 are amplified also. These known side-products were not considered as errors. Alignments were examined for pseudogene evaluation.

As an error correction tool Acacia [21] was used with default parameters, the improved sequences were investigated with respect to the previous error results.

### Statistics

A series of Perl 5.10.0 scripts (The Perl Foundation, Walnut, CA, USA) was used for variant data extraction, mapping of quality values to variant positions and assessment of read qualities and homopolymer runs. R 2.14.2 (2012-02-29) [36] was used for graphics generation and statistical tests. For averaging quality scores they were translated to error rates, then averaged and transferred back to average quality scores.

### Availability of supporting data

Sequence information is available at NCBI’s SRA database, accession number SRP020222.

## Abbreviations

HLA: Human leukocyte antigens; NGS: Next-generation sequencing; SBT: Sequence-based typing; DNA: Deoxyribonucleic acid; PTP: PicoTiterPlate; CCD: Charge-coupled device; PCR: Polymerase chain reaction; AVA: Amplicon Variant Analyzer; EFI: European Federation for Immunogenetics.

## Competing interests

The authors declare that they have no competing interests.

## Authors’ contributions

NN performed bioinformatic and statistical analyses and wrote the manuscript. JP, MD and CG designed research and critically reviewed the manuscript. SS and KH performed GS Junior sequencing. All authors read and approved the final manuscript.

## Supplementary Material

Additional file 1 **Error positions per amplicons.** Additional documentation is provided in portable document format (.pdf), including plots of frequent error positions per amplicon. Click here for file
