# Supplementary material for: Routine performance and errors of 454 HLA exon sequencing in diagnostics
Source: BMC Bioinformatics. 2013 Jun 3;14:176. doi: 10.1186/1471-2105-14-176 (PMC3679934; doi:10.1186/1471-2105-14-176)
Supplement: Additional file 1 — Error positions per amplicons. Additional documentation is provided in portable document format (.pdf), including plots of frequent error positions per amplicon. [file 1471-2105-14-176-S1.pdf]

Additional file 1

Frequency of errors occurring at a specified read position (in relation to the coverage) in total (white) and without homopolymer association (gray) are plotted per amplicon (exon). Bars are unstacked, homopolymer proportions plotted to the front. The boxplots below display the quartiles of errors across read positions (total). Errors at the first positions are very infrequent, furthermore, this section of sequence is located outside the analyzed exon.

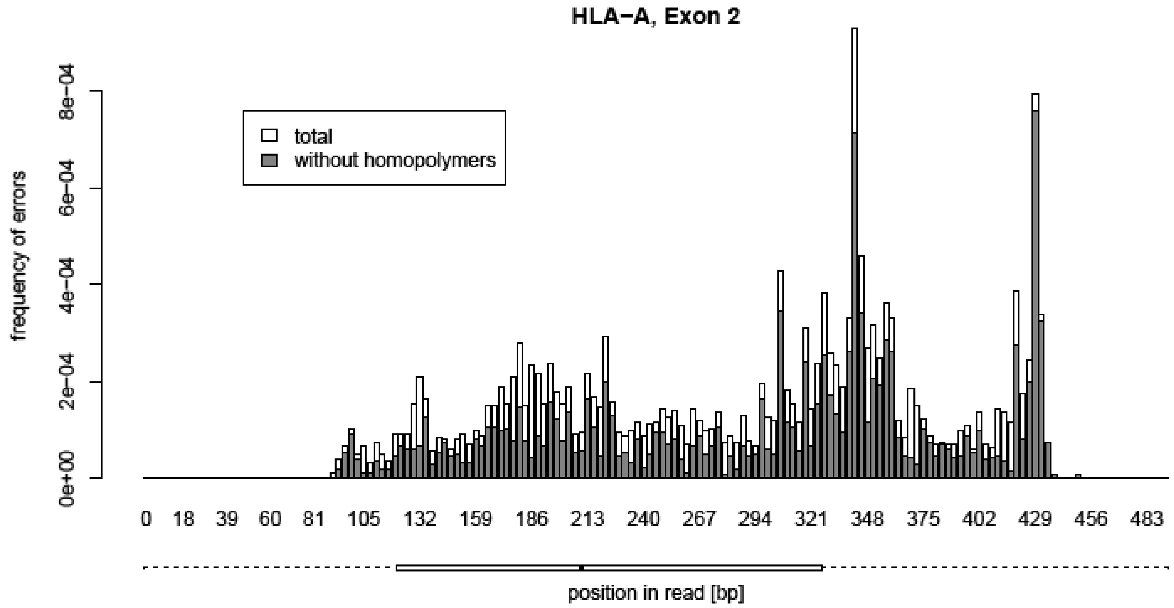

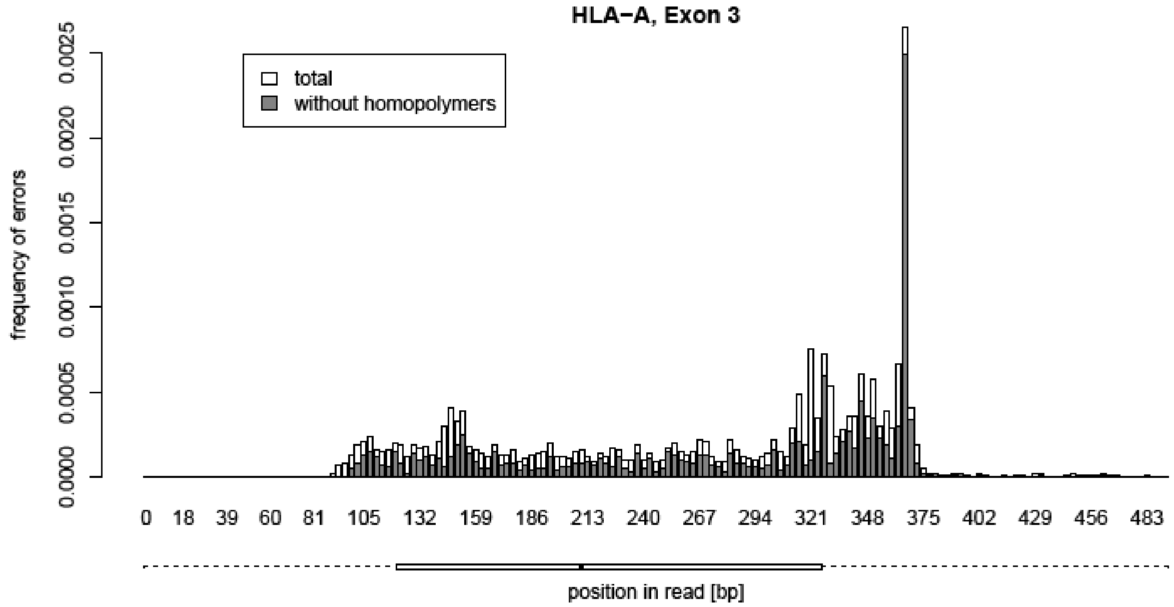
HLA−A, Exon 3
total
without homopolymers
frequency of errors
0.0000
0.0005
0.0010
0.0015
0.0020
0.0025
0 18 39 60 81 105 132 159 186 213 240 267 294 321 348 375 402 429 456 483
position in read [bp]

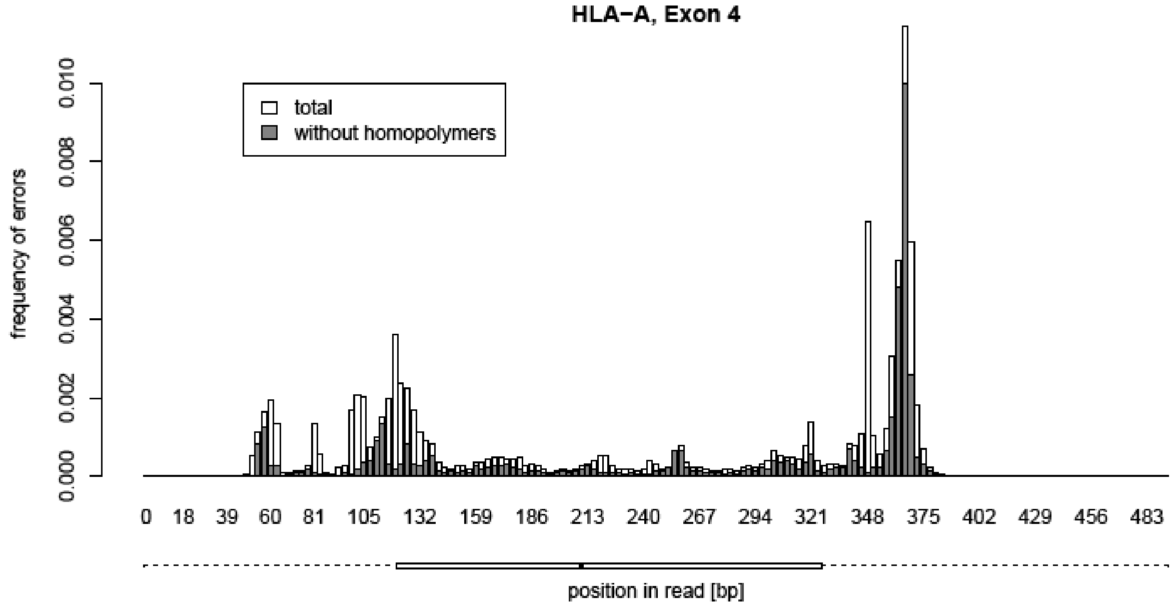
HLA−A, Exon 4
total
without homopolymers
frequency of errors
0.000
0.002
0.004
0.006
0.008
0.010
0 18 39 60 81 105 132 159 186 213 240 267 294 321 348 375 402 429 456 483
position in read [bp]

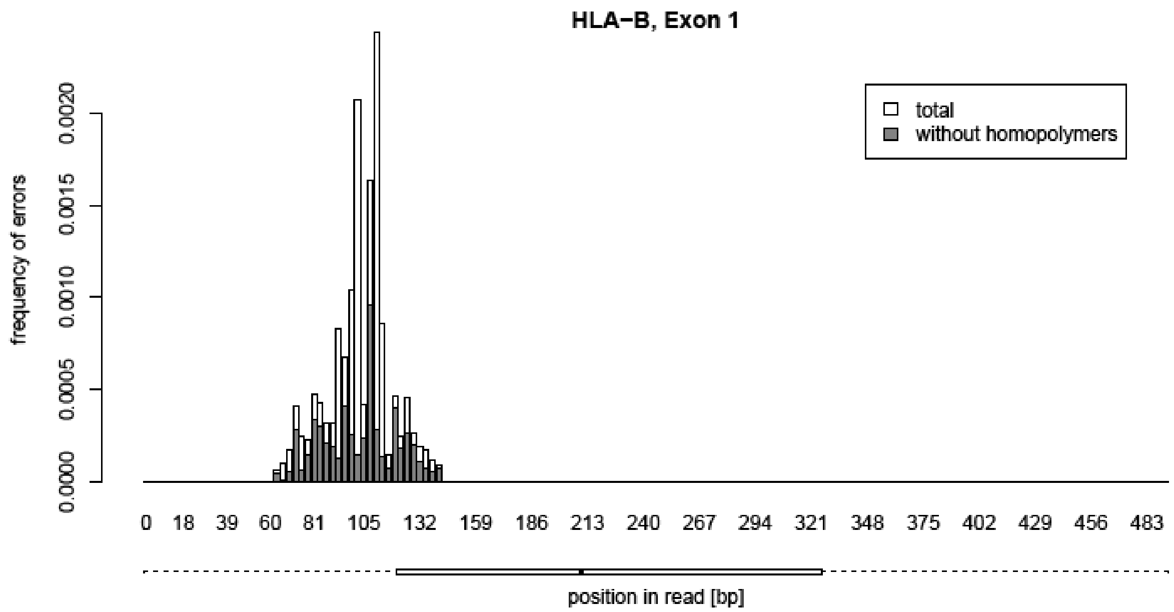
HLA−B, Exon 1
total
without homopolymers
frequency of errors
0.0000
0.0005
0.0010
0.0015
0.0020
0 18 39 60 81 105 132 159 186 213 240 267 294 321 348 375 402 429 456 483
position in read [bp]

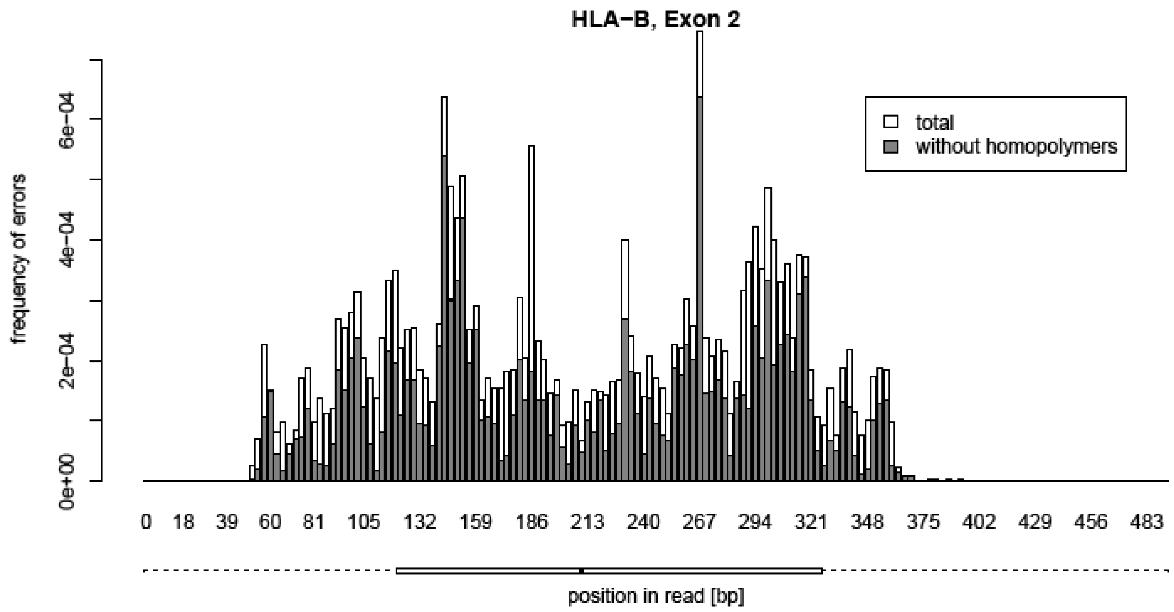
HLA−B, Exon 2
total
without homopolymers
frequency of errors
0e+00
2e−04
4e−04
6e−04
0 18 39 60 81 105 132 159 186 213 240 267 294 321 348 375 402 429 456 483
position in read [bp]

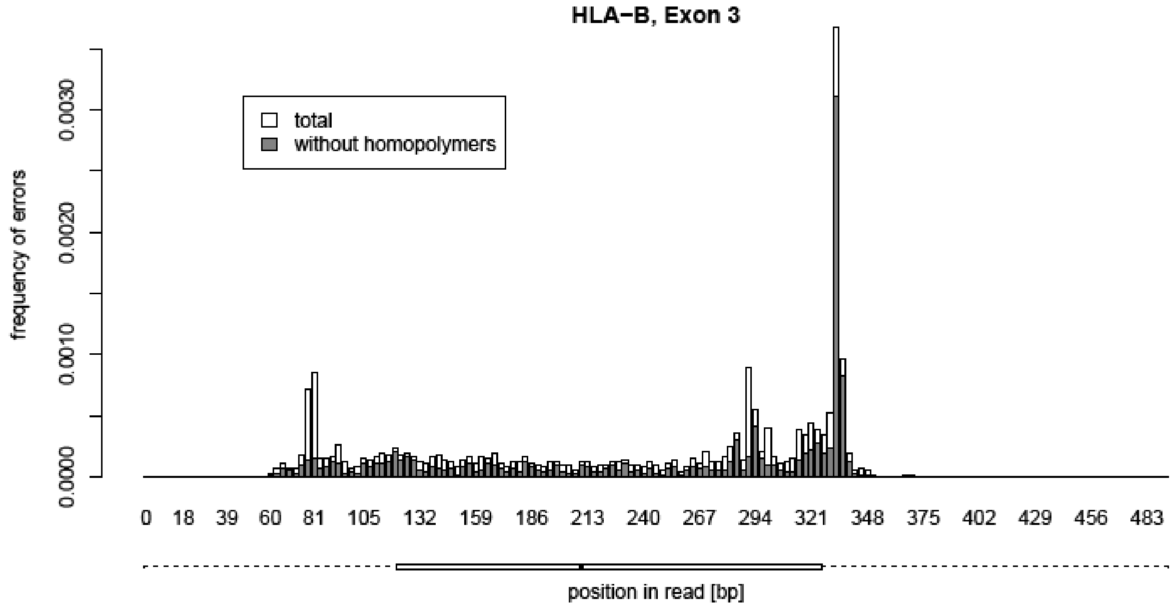
HLA−B, Exon 3
total
without homopolymers
frequency of errors
0.0000
0.0010
0.0020
0.0030
0 18 39 60 81 105 132 159 186 213 240 267 294 321 348 375 402 429 456 483
position in read [bp]

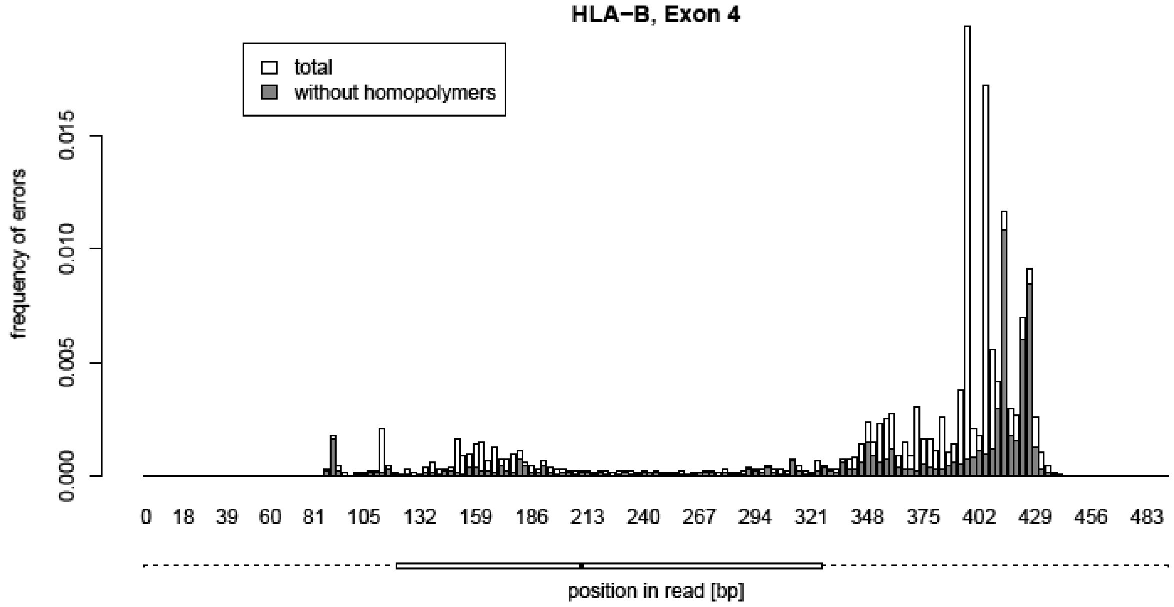
HLA−B, Exon 4
total
without homopolymers
frequency of errors
0.000
0.005
0.010
0.015
0 18 39 60 81 105 132 159 186 213 240 267 294 321 348 375 402 429 456 483
position in read [bp]

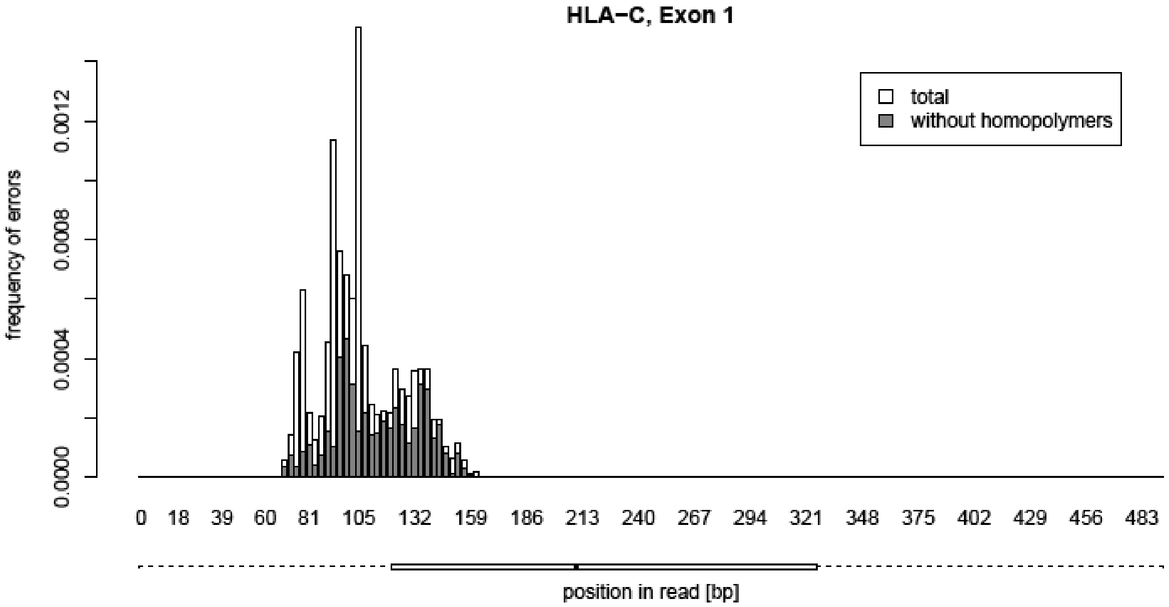
HLA−C, Exon 1
total
without homopolymers
frequency of errors
0.0000
0.0004
0.0008
0.0012
0 18 39 60 81 105 132 159 186 213 240 267 294 321 348 375 402 429 456 483
position in read [bp]

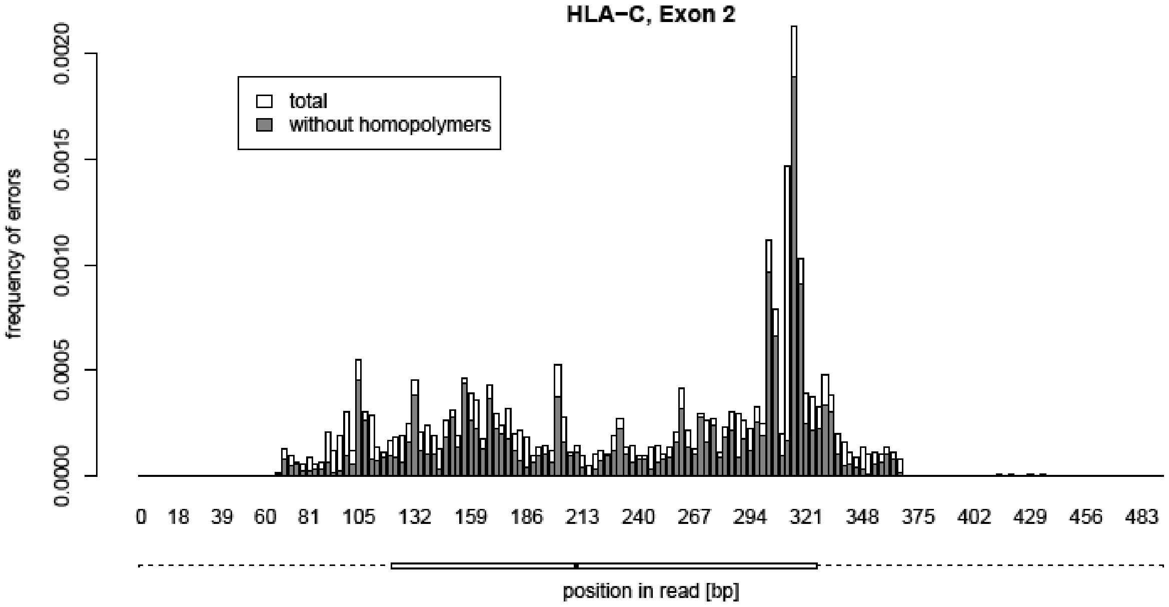
HLA−C, Exon 2
total
without homopolymers
frequency of errors
0.0000
0.0005
0.0010
0.0015
0.0020
0 18 39 60 81 105 132 159 186 213 240 267 294 321 348 375 402 429 456 483
position in read [bp]

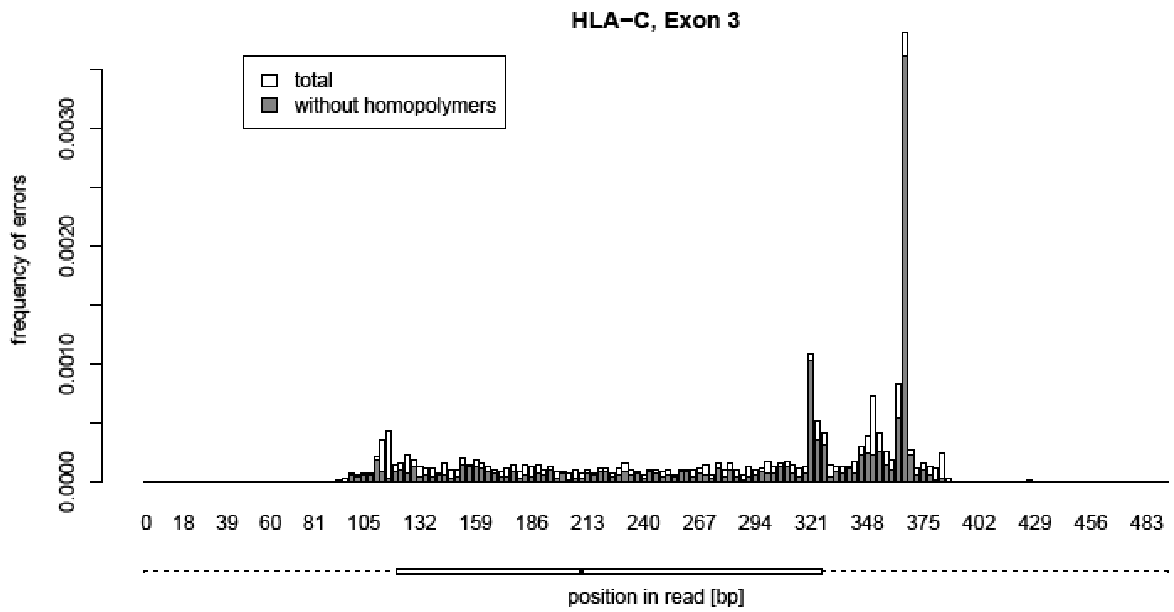

HLA−C, Exon 3
total
without homopolymers
frequency of errors
0.0000
0.0010
0.0020
0.0030
0 18 39 60 81 105 132 159 186 213 240 267 294 321 348 375 402 429 456 483
position in read [bp]

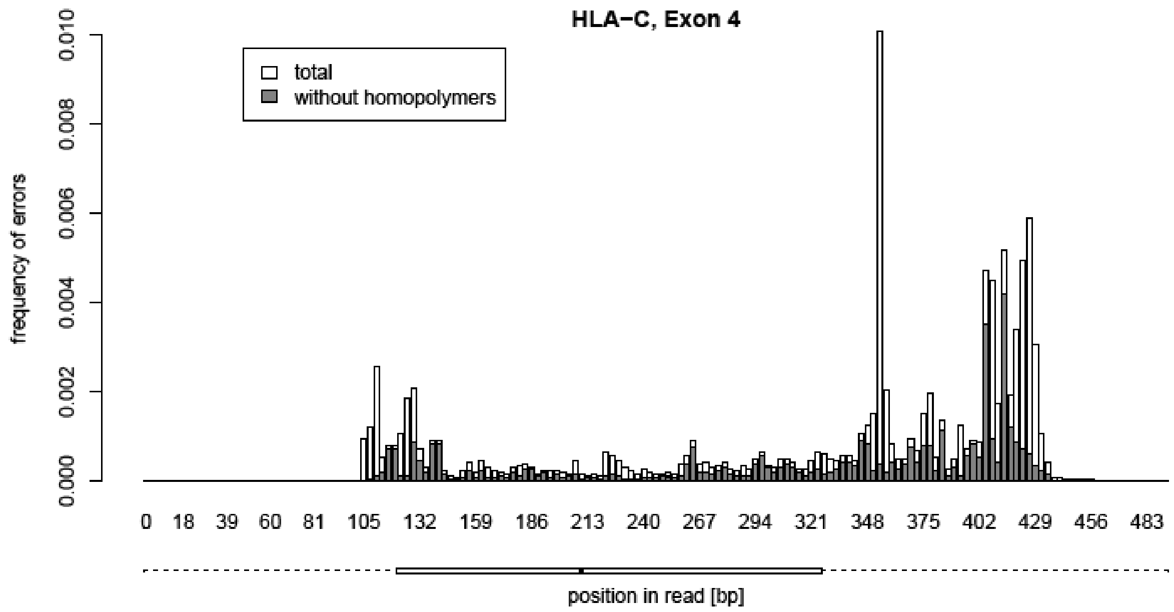

HLA−C, Exon 4
total
without homopolymers
frequency of errors
0.000
0.002
0.004
0.006
0.008
0.010
0 18 39 60 81 105 132 159 186 213 240 267 294 321 348 375 402 429 456 483
position in read [bp]

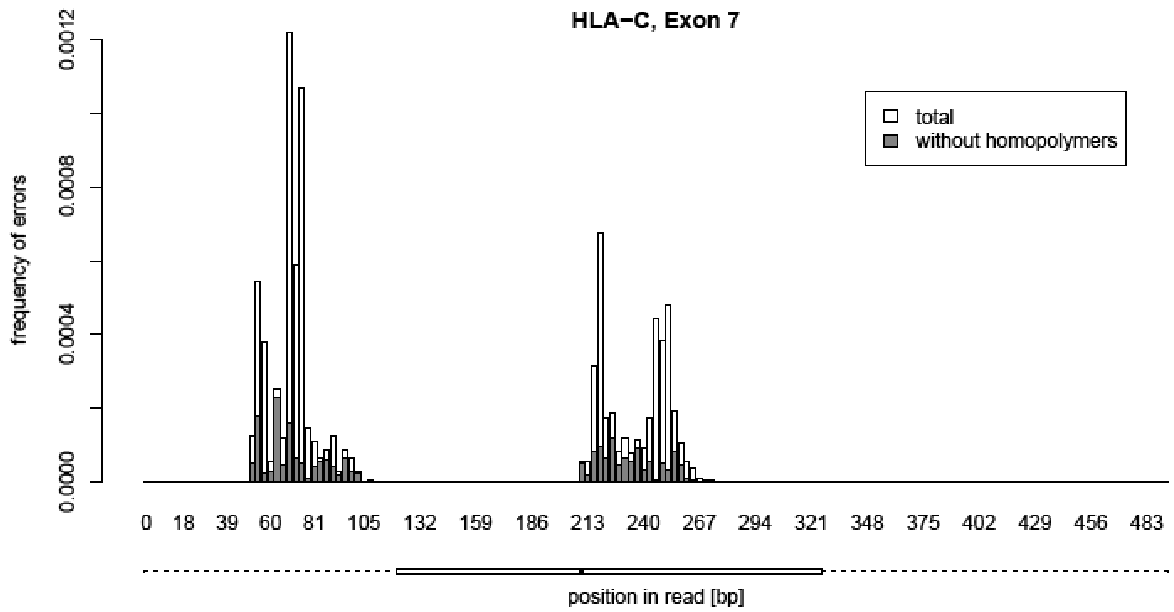
HLA−C, Exon 7
total
without homopolymers
frequency of errors
0.0000
0.0004
0.0008
0.0012
0 18 39 60 81 105 132 159 186 213 240 267 294 321 348 375 402 429 456 483
position in read [bp]

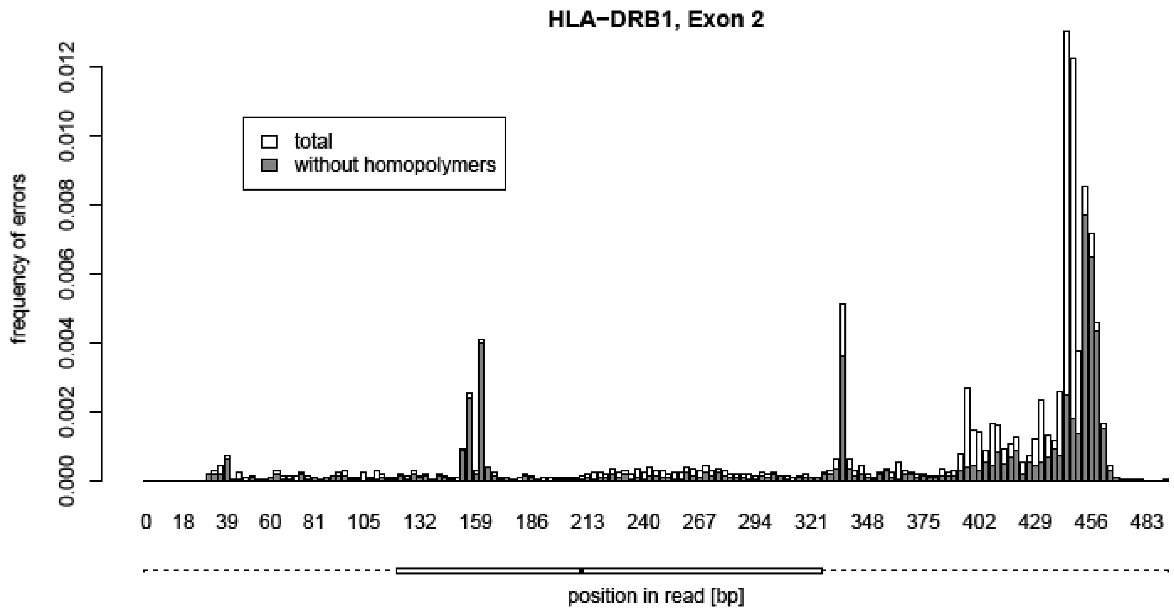
HLA−DRB1, Exon 2
total
without homopolymers
frequency of errors
0.000
0.002
0.004
0.006
0.008
0.010
0.012
0 18 39 60 81 105 132 159 186 213 240 267 294 321 348 375 402 429 456 483
position in read [bp]

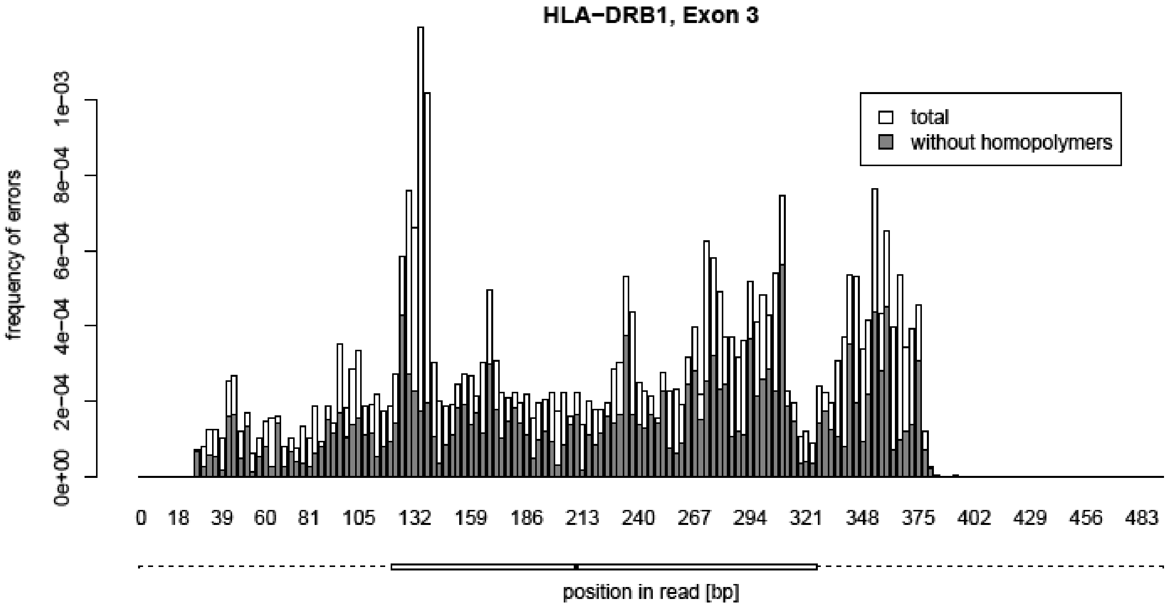
HLA−DRB1, Exon 3
frequency of errors
0e+00
2e−04
4e−04
6e−04
8e−04
1e−03
total
without homopolymers
0 18 39 60 81 105 132 159 186 213 240 267 294 321 348 375 402 429 456 483
position in read [bp]

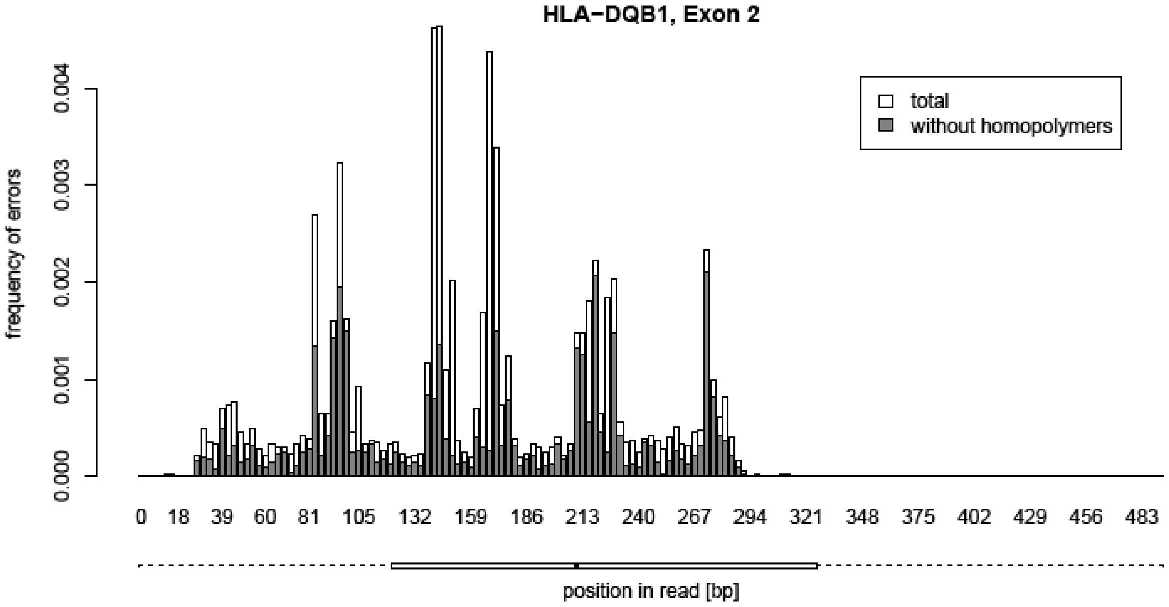
HLA−DQB1, Exon 2
frequency of errors
0.000
0.001
0.002
0.003
0.004
total
without homopolymers
0 18 39 60 81 105 132 159 186 213 240 267 294 321 348 375 402 429 456 483
position in read [bp]

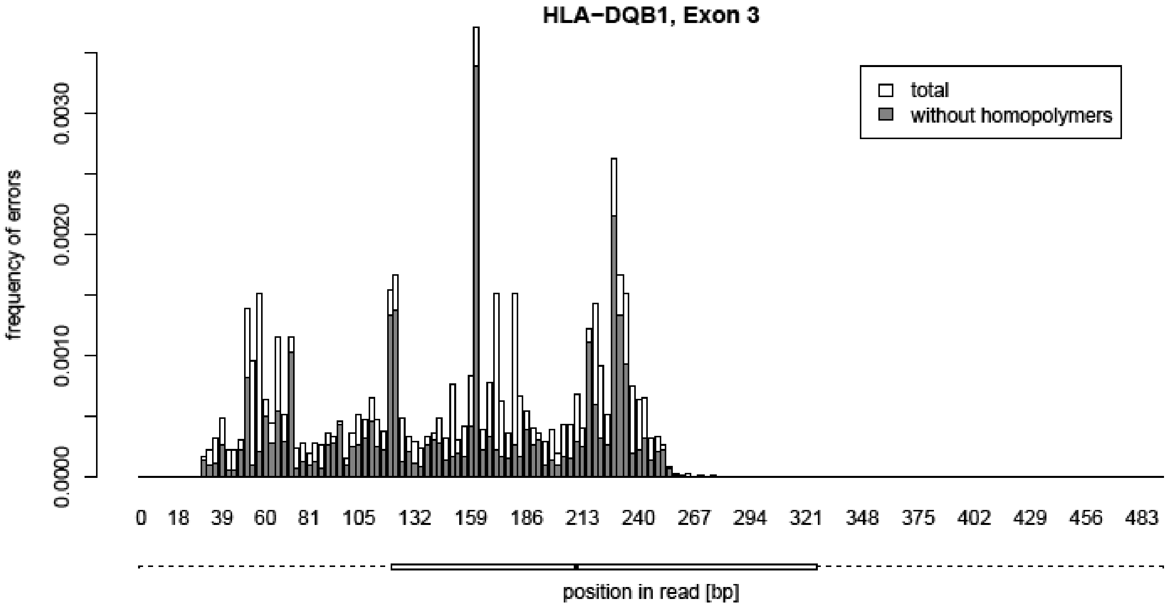
HLA−DQB1, Exon 3
total
without homopolymers
frequency of errors
0.0000
0.0010
0.0020
0.0030
0 18 39 60 81 105 132 159 186 213 240 267 294 321 348 375 402 429 456 483
position in read [bp]

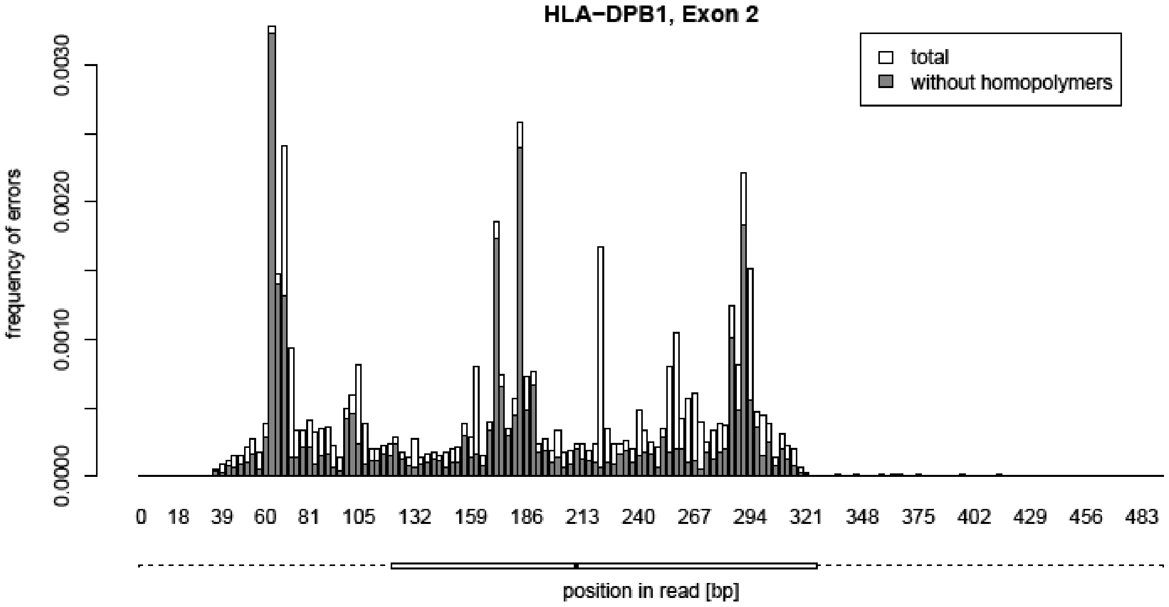
HLA−DPB1, Exon 2
total
without homopolymers
frequency of errors
0.0000
0.0010
0.0020
0.0030
0 18 39 60 81 105 132 159 186 213 240 267 294 321 348 375 402 429 456 483
position in read [bp]
